# Quality of depression assessments in child and adolescent psychiatry: Findings from a nationwide Swedish outpatient medical record review

**DOI:** 10.1177/13591045251341919

**Published:** 2025-06-24

**Authors:** Susanne Remvall, Anna Helena Elisabeth Santesson, Martin Bäckström, Björn Hofvander, Håkan Jarbin

**Affiliations:** 1 Department of Child and Adolescent Psychiatry, 59565 Skåne University Hospital, Sweden; 2 Unit for Child and Adolescent Psychiatry, Department of Clinical Sciences Lund, 5193 Lund University, Sweden; 3 Department of Child and Adolescent Psychiatry, 293183 Region Halland, Sweden; 4 Unit for Psychology, Department of Social Sciences, 5193 Lund University, Sweden; 5 Department of Forensic Psychiatry, 59565 Skåne University Hospital, Sweden; 6 Lund Clinical Research on Externalizing and Developmental Psychopathology, Department of Clinical Sciences Lund, 5193 Lund University, Sweden

**Keywords:** Child, adolescent, depression, depressive disorders, diagnosis, assessment, quality, indicator, predictor, principal component

## Abstract

**Background:** Early-onset depression is an increasing concern, contributing to long-term disability and suicide. Diagnostic assessments are essential for effective treatment. However, research suggests that Child and Adolescent Psychiatry practices remain inadequate. Knowledge of healthcare processes is limited, with no consensus on conceptualising or measuring quality. This study aimed to investigate depression assessments in Swedish outpatient Child and Adolescent Psychiatry, including associations with predictors and diagnostic timeliness. **Methods:** Medical records (*n* = 284) from patients aged 8–17 with depression were collected from 10 services. Quality indicators for depressive symptoms, suicidality, comorbidities, and functioning were developed from guidelines. Indicator occurrences were assessed, summarised into components, and analysed using regression and correlations. **Results:** Indicator occurrences ranged from 8% to 84%, averaging 49%. Documentation varied considerably for risk aspects (57%). Distinct depressive characteristics (63%) occurred nearly twice as often as subtler symptoms (35%). Comorbidities (13%–22%) were rarely documented, whereas functioning and life situation (69%) were well-recorded. Predictors explained up to 28% of variance, with unidentified service-related factors explaining 10%. Better documentation weakly correlated with earlier diagnoses. **Conclusions:** Findings indicate the need for guideline implementation and further investigation into assessment inequities. Improving quality might promote earlier diagnoses. The indicators may be applicable in similar settings.

## Plain Language Summary

**How is Depression Assessed in Young People in Sweden? Insights from Medical Records** **Why this study was done: **Depression in young people is becoming more common and is a major cause of long-term health problems and suicide among young people. Accurate assessments are essential for effective treatment, but research shows that current practices often fall short of best standards. There is limited knowledge on how to improve these assessments, and no agreement on how to measure their quality. **What was done: **This study investigated the quality of depression assessments in Swedish outpatient psychiatry for children and adolescents. Researchers reviewed medical records of 284 patients aged 8–17 diagnosed with depression. The study evaluated how well clinics documented symptoms of depression, suicidality, other mental health issues, and patient functioning, using quality measures based on clinical guidelines. Documentation quality was analysed in relation to patient and clinical characteristics, but also how quickly diagnoses were made. **What was found: **Documentation quality varied widely, especially for risk assessments. On average, only half of the recommended areas were properly documented. Depressed mood, suicidal thoughts, and sleep disturbance, as well as patient functioning, were the best-documented areas. However, less obvious depressive symptoms, like loss of interest or pleasure (anhedonia), and evaluations of other mental health conditions were often overlooked. Use of self-report tools was linked to better documentation, but unknown clinic-related factors also influenced the recordings. **What this means: **These findings highlight the need for improved adherence to clinical guidelines and additional training for healthcare providers. Enhancing how depression assessments are documented could lead to more effective evaluations and earlier diagnoses, thereby improving treatment outcomes. The quality measures developed in this study may also be useful for clinics elsewhere to enhance their assessment practices.

## Introduction

Early-onset depression is a growing public health concern (Herrman et al., 2022), with one in five experiencing major depressive disorder before adulthood (Shorey et al., 2021). Despite knowledge of evidence-based interventions (NICE, 2019; Walter et al., 2023), many children and adolescents with depression do not receive optimal care due to limited and unequally distributed resources (Kruk et al., 2018; Moitra et al., 2022; Mora Ringle et al., 2019; Socialstyrelsen, 2019; WHO, 2021). Depression is a major contributor to the increasing global burden of disease (Ferrari et al., 2022), with suicide as its most fatal consequence and a leading cause of death among young people (Moitra et al., 2021). Depressive disorders are heritable and often coexist with other psychiatric conditions, requiring special considerations (Avenevoli et al., 2015; Thapar et al., 2022). They increase the risk of future somatic, psychosocial, and psychiatric problems (Clayborne et al., 2019; Johnson et al., 2018; Leone et al., 2021), severely affecting quality of life, functioning, and development, leading to long-term disability and substantial societal losses (Bodden et al., 2018; Kieling et al., 2011; Wickersham et al., 2021).

High-quality diagnostic assessments are essential for evidence-based healthcare and a prerequisite for appropriate and successful treatment (Jensen-Doss & Weisz, 2008). For adolescents, self-reports are vital for assessing and treating internalising symptoms like depression and suicidality (De Los Reyes et al., 2015; Hawton et al., 2022; Housby et al., 2021; Krause et al., 2023; Lewis et al., 2014). However, clinicians tend to underdiagnose depression in young people, inadequately assessing diagnostic criteria and failing to consider comorbidities or alternative explanations (Jensen-Doss et al., 2014; Nakash et al., 2015). Although patient and family characteristics have been associated with diagnostic reliability and guideline adherence, clinical resources and systematic approaches such as Longitudinal Expert All Data (LEAD) appear more essential (Jensen-Doss et al., 2014; Mora Ringle et al., 2019), while the use of fully structured diagnostic instruments remains debated (Duncan et al., 2019; Sayal et al., 2025). Additionally, attitudes towards the utility of diagnoses, diagnostic instruments, and clinical practice guidelines vary across healthcare professions, affecting diagnostic reliability (Jensen-Doss & Hawley, 2011; Santesson et al., 2024).

***Mental healthcare quality*****.** Clinical practice guidelines aim to improve healthcare quality, but implementation is complex and often requires multifaceted strategies (Wensing et al., 2020). The ultimate goal is improved patient outcomes, though these outcomes are often affected by factors other than healthcare quality and do not provide direct information on how to improve quality (Lilford et al., 2007). Therefore, healthcare process measures are considered more suitable for assessing healthcare quality (Wensing et al., 2020). When valid and reliable, such measures reveal relationships between structure, processes, and patient outcomes (Kilbourne et al., 2018). However, efforts to identify universal psychiatric quality indicators have yielded few measures applicable to outpatient depression care for children and adolescents (Iyer et al., 2016).

**Figure 1.**
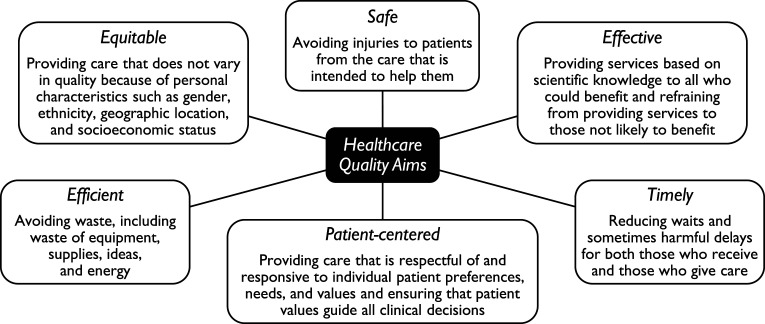
Six aims for healthcare quality improvement (IOM, 2001).

The Institute of Medicine (IOM) established six aims for healthcare quality improvement (IOM, 2001), as detailed in Figure 1. Consistent with the World Health Organization (WHO) quality dimensions (WHO, 2006), they emphasise the importance of evidence-based assessments. Their implementation has however been hindered by limited scientific evidence (Quinlan-Davidson et al., 2021). Challenges include insufficient knowledge of care processes and a lack of consensus on conceptualising and measuring quality within frameworks (Kilbourne et al., 2018; Quinlan-Davidson et al., 2021; Skokauskas et al., 2019; Williams & Beidas, 2019). Recent updates to implementation frameworks stress the need for new approaches and evolving methods (Damschroder et al., 2022; Skivington et al., 2021), alongside clear targets, mechanisms, outcomes, and standard measures (Williams & Beidas, 2019). Empirical approaches for constructing, aggregating, and validating quality measures are increasingly emphasised (Kilbourne et al., 2018; Wensing et al., 2020).

Medical Record Review (MRR) allows detailed proxy measurement of care processes and is widely used in healthcare quality research (Gearing et al., 2006; Vassar & Holzmann, 2013). It enables investigations of phenomena unsuitable for randomised prospective studies but is less effective for processes that are not commonly documented (Worster & Haines, 2004). Detailed process measurements are difficult to discern through administrative systems, and focusing on single indicators risks unintended displacement effects, as healthcare providers may deprioritise other processes (Kilbourne et al., 2018; Wensing et al., 2020). Therefore, better data sources for assessing healthcare performance have been called for (Socialstyrelsen, 2019). Yet, MRR validity and reliability depend on the comparability of measures and analytical methods (Gearing et al., 2006; Vassar & Holzmann, 2013).

***Depression assessment quality*****.** A few, heterogenous studies have assessed outpatient depression care in children and adolescents using MRR (Ellis et al., 2019; Hermens et al., 2015; Kramer et al., 2008; Pile et al., 2020; Zima et al., 2005). Previous studies applied different consensus methods to develop quality indicators, which were rarely similarly defined. All used binary opportunity indicator assessment, presenting results as proportional occurrences. Three studies based their indicators on clinical guidelines (Ellis et al., 2019; Hermens et al., 2015; Pile et al., 2020). While suicide risk assessment was a common indicator, no consensus on other assessment indicators was found. Overall, these studies demonstrated depression assessment quality indicator occurrences above 50%: ∼90% for risk factors (Pile et al., 2020) and suicide risk (Zima et al., 2005); ∼85% for family psychiatric history (Pile et al., 2020; Zima et al., 2005); ∼80% for personal/interpersonal conditions (Ellis et al., 2019; Zima et al., 2005), family climate (Ellis et al., 2019), and parent reports (Zima et al., 2005); ∼80% for function and depression severity (Ellis et al., 2019; Hermens et al., 2015); ∼75% for substance abuse (Kramer et al., 2008; Zima et al., 2005), ∼70% for self-harm and suicide risk (Ellis et al., 2019; Kramer et al., 2008), and self-rating usage (Pile et al., 2020); ∼60% for school reports, aggressive behaviour (Zima et al., 2005), and considering other explanations (Ellis et al., 2019). None of these studies used specific depressive symptoms or comorbidities as quality indicators, nor did they utilise continuous scales, which have been recently recommended (Damschroder et al., 2022). The lack of empirical approaches for summarising indicators and alignment with healthcare quality frameworks highlights persistent knowledge gaps in quality measurement and conceptualisation in this area.

### Objectives

The primary objective was to investigate the quality of depression assessments in Swedish outpatient child and adolescent psychiatry in relation to clinical guideline standards. Secondary objectives, aligned with the IOM quality aims, were to analyse associations between assessment quality and demographic or clinical predictors, as well as time until diagnosis.

## Method

This cross-sectional study was part of a Swedish multicentre research programme that utilises mixed methods to assess the nationwide implementation of a clinical practice guideline for depression (Jarbin et al., 2014). Additional information about the implementation programme is provided in the Supplemental information section.

### Setting

**Figure 2.**
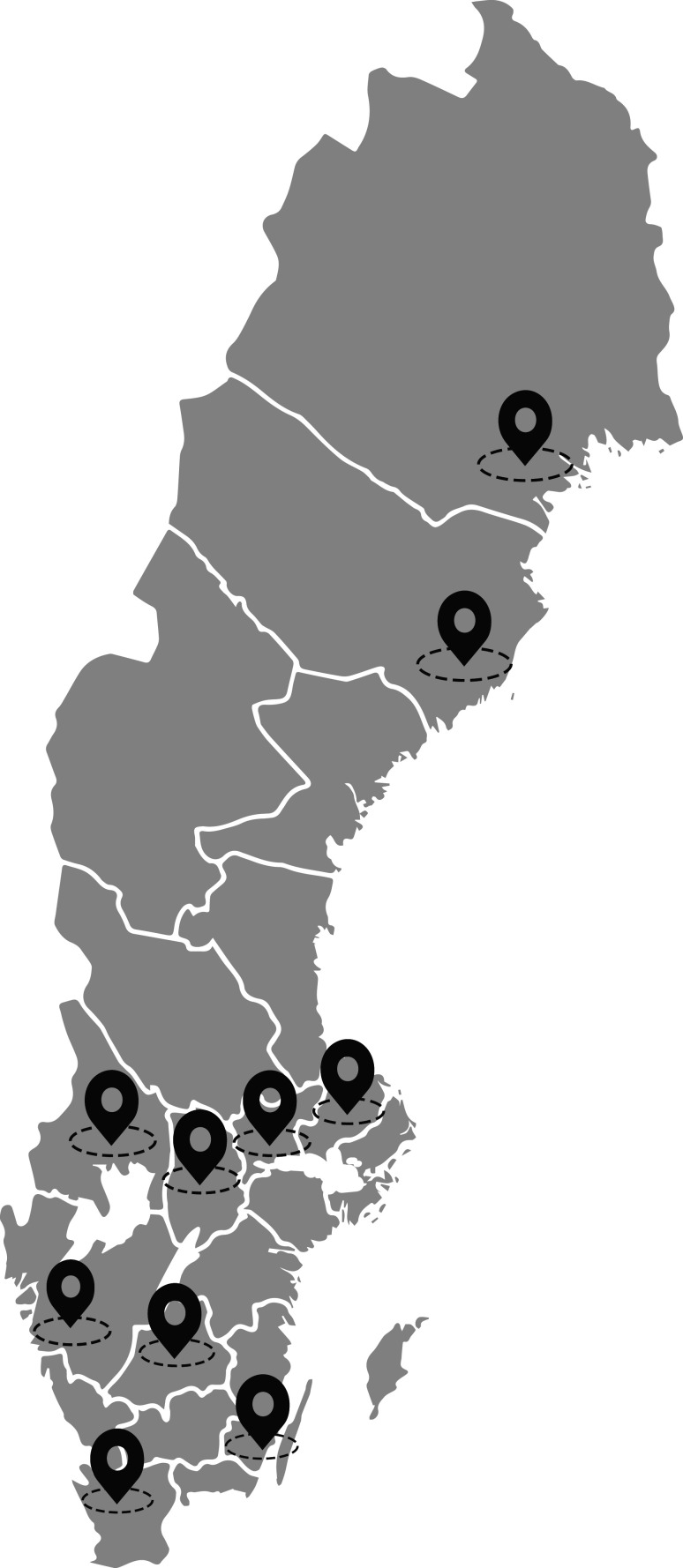
Sweden – regions and locations of participating services (*n* = 10).

The healthcare system in Sweden is mainly publicly funded, with self-governing, tax-funded regions responsible for healthcare delivery, shown in Figure 2. In 2014, the 31 publicly funded Swedish outpatient CAMHS, serving an average of 64,000 children annually (range: 29,000–450,000), were invited to participate in the programme. Depressive disorders were typically treated within CAMHS, where they accounted for 8% of all diagnoses (Socialstyrelsen, 2014). CAMHS professionals included psychologists, social workers, nurses, psychiatrists, and non-specialist doctors. Clinical evaluations were primarily based on the Diagnostic and Statistical Manual of Mental Disorders, Fifth Edition (DSM-5) (APA, 2013), though diagnoses were coded according to the International Statistical Classification of Diseases and Related Health Problems – Tenth Revision (ICD-10) (WHO, 2004).

### Participants

The first 10 services agreeing to participate, shown in Figure 2, were included in this study. Each served around 65,000 children annually (range: 41,000–125,000), collectively covering about one-third of Sweden’s children and adolescents. Their catchment areas, as well as geographical and demographic conditions, were similar to those of non-participating services. Participating services contributed 40 consecutive medical records, coded F32–34 per ICD-10 (WHO, 2004), starting from 1 January 2014 and covering up to six months. Patient eligibility criteria included: a depression diagnosis (major or recurrent depressive episodes and other depressive disorders), a first outpatient session in 2014, age up to 17, and at least two outpatient sessions within the first two months.

***Study size*****.** A power analysis using one-way analysis of variance (ANOVA) tested the null hypothesis of identical population means across services (*n* = 10). At least 30 medical records per service were estimated to detect moderate outcome variance. The total sample size (*n* = 300), with an effect size of .1 Eta-squared and a significance level of α = .001, yielded a power of 0.863 (based on noncentral *F*-distribution), sufficient to detect small differences in large subgroups while allowing for some exclusions.

### Procedure

The IOM healthcare quality aims (IOM, 2001), presented in Figure 1, provided the framework for quality conceptualisation, methodology, and interpretation. The primary objective aligned with the aims of Safe, Efficient, Effective, and Patient-centered care, while secondary objectives addressed Equitable and Timely care. Assessment quality was measured via MRR using quality indicators based on key recommendations from the Swedish Association of Child and Adolescent Psychiatry clinical guideline for depression (Jarbin et al., 2014). A structured protocol was developed for data abstraction from medical records, with standardised paper forms used before transcription into a dataset for statistical analyses. The instrument was piloted on records from a non-participating service, and the principal investigator compiled a procedural manual and video instruction. Training included a thorough review of variables, the procedural manual, and the abstraction instrument.

Initial data abstraction was performed by two research assistants (medical and psychology students) who were familiar with participant identification numbers but had no other involvement in the study and were unaware of the purpose of the MRR. Coding validity and reliability were monitored and verified by the investigators, who also determined exclusions. To ensure accuracy, one investigator (child and adolescent psychiatrist) re-reviewed all medical records. Interrater reliability was assessed by coding every sixth record, analysed using a type C Intraclass Correlation Coefficient (ICC) with a two-way mixed-effects model (DeVellis, 2017).

***Ethical considerations*****.** Procedures complied with Swedish institutional and national ethical standards, the 1964 Helsinki Declaration, and its amendments. Ethical approval was granted, with details available in the Supplemental information section. Informed consent was obtained from patients and caregivers, who received study information and had the opportunity to opt out. Medical records were de-identified for pseudo-anonymisation, and results were reported only at the group level.

### Measures

The validity and reliability of quality indicators were ensured through a two-stage operationalisation process, following recommended methods (Gearing et al., 2006; Vassar & Holzmann, 2013; Worster & Haines, 2004). The guideline was developed using a modified RAND Appropriateness Method (Fitch et al., 2001), referencing Swedish National Board of Health and Welfare guidelines (Socialstyrelsen, 2010), international guidelines, and meta-analyses. Content validity was supported by DSM-5 (APA, 2013) and the Kiddie Schedule for Affective Disorders and Schizophrenia (K-SADS), validated in a Swedish study (Jarbin et al., 2017). A systematic literature review confirmed reasonable agreement with the indicators.

Quality indicator occurrences in medical records were assessed using a three-step quality scale: 0 = missing/inadequate, 1 = partly present/somewhat adequate, and 2 = present/fully adequate. Separate codes were used for non-applicable cases and negated issues considered present/fully adequate when applicable. Indicators included: depressive symptoms per DSM-5 (APA, 2013), course of depression (episodicity), family psychiatric history, differential diagnoses or comorbidities to consider (mania/hypomania – bipolar disorder; anxiety disorders/ Obsessive Compulsive Disorder [OCD]; disruptive behaviour disorders; Attention Deficit Hyperactivity Disorder [ADHD]), functioning and life situation – including need for support and maintaining or protective factors (peer relations; schooling; family climate), substance abuse (applied to teenagers), non-suicidal self-injury, suicide risk assessments – graded (low; moderate; high) and structured with risk factors (if moderate or high risk), and diagnostic statement including depression severity and consideration of other explanations. Descriptive information for independent variables, aligned with the IOM framework (IOM, 2001), was obtained from medical records and administrative systems: patient age and sex, proportion of joint sessions with patient and caregiver, use of depression self-rating scales, diagnostician’s profession (Psychiatrist, including non-specialist doctors, at least once; Psychologist, if no psychiatrist; Other, if no psychiatrist/psychologist), service (site), and time (weeks) until diagnosis. Coding requirements for all variables are detailed in Supplemental Table S1.

### Statistical analyses

The primary measure was the occurrence of quality indicators in medical records, grouped into components reflecting the IOM aims of Safe, Efficient, Effective, and Patient-centered care. Secondary measures for Equitable care included coefficients of determination (*R*^2^) and parameter estimates from multiple linear regression analyses, detecting associations between quality and predictors. For Timely care, correlations between quality and time until diagnosis were analysed using Pearson product-moment coefficients (*r*), with time transformed into square root weeks due to positive skewness.

Principal Component Analysis using polychoric correlations identified documentation patterns by empirically grouping quality indicators that frequently co-occurred in medical records. Parallel analysis suggested extracting five components, rotated using the Promax criterion. An additional component was added for better interpretability after inspection. Quality indicators and components underwent standard parametric analyses for proportions, with binomial 95% confidence intervals (95% *CI*), distributions, and dispersion measures. Internal consistency for all quality indicators, forming the Indicator Index, was analysed using Cronbach’s coefficient α. Missing data (six values) were imputed after raw data review.

Preliminary analyses revealed uneven distributions of clinical characteristics, prompting regression analyses to account for group heterogeneity (Tabachnick & Fidell, 2013). To reduce outlier influence, patients aged 12 and below were grouped, and other predictors were effect-coded as binary indicators. Variables were examined for normality, linearity, heteroscedasticity, multicollinearity, and interaction effects. Site predictors showed collinearity with self-rating and profession, leading to the use of two models, with site predictors omitted in the second. As no significant interactions were found, these terms were excluded from the final models. Performance analyses included *R*^2^, *F*-tests for ANOVA, tolerance, and variance inflation factor values, with parameter estimates evaluated solely for the second model. A significance level of α = .05 was used throughout, with Bonferroni-corrected levels provided in table notes.

***Statistical software and reporting guideline*****.** The Principal Component Analysis was performed using R (RCoreTeam, 2024), package psych (Revelle, 2024). All other statistical analyses used the Statistical Package for Social Science (IBM, 2016). The reporting of this study was guided by the Strengthening the Reporting of Observational Studies in Epidemiology (STROBE) statement (von Elm et al., 2007).

## Results

**Figure 3.**
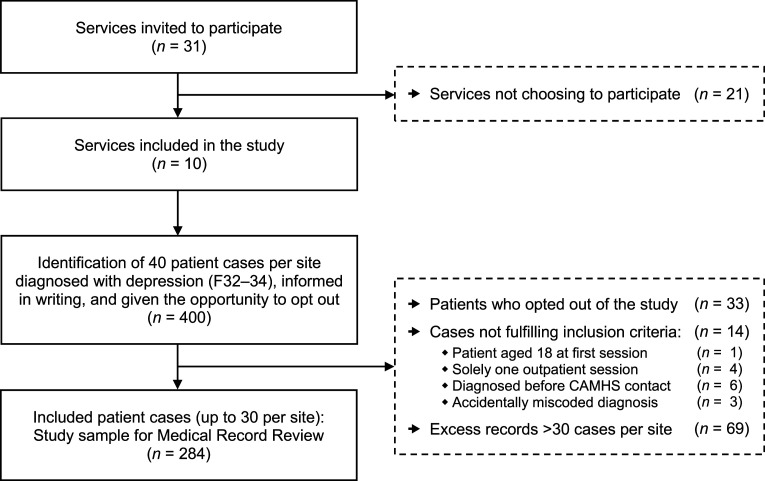
Flow diagram of inclusion process for participating services (*n* = 10) and patient cases (*n* = 284).

The inclusion process and reasons for non-participation are shown in Figure 3. Of the 400 selected cases, 33 patients (8%) from six services opted out. Among the remaining 367 eligible records, 14 from four services were excluded for not meeting the inclusion criteria. Once each site reached the target of 30 cases, 69 additional records from seven services were excluded from the analyses. Three services could not provide 30 cases each, resulting in a final sample of 284 records.

### Sample characteristics

**Figure 4.**
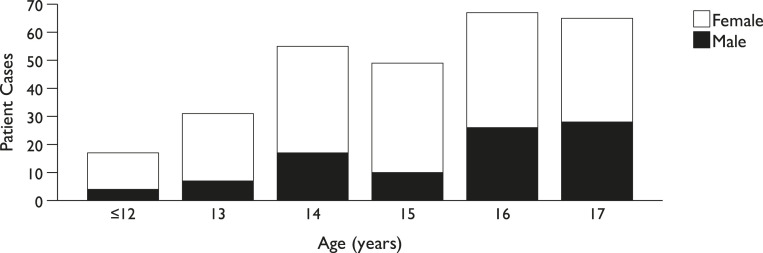
Distribution of patient characteristics (*n* = 284).

The typical case involved a 16-year-old female patient with a moderate proportion of joint sessions, no self-reported ratings, at least one psychiatrist session, and a depression diagnosis established within the first week of CAMHS contact. Demographic and clinical details of the study participants are shown in Table 1. The average patient age was 15.1 years (*SD* = 1.6). Female patients (68%) were twice as common as males (32%), with an even age distribution, as illustrated in Figure 4. A caregiver participated in at least one assessment session in all cases.

**Table 1.** Descriptive Characteristics of Study Participants (*n* = 284).

Characteristics	Specifications	*N*	%
Patient age (years)	≤12	17	6
	13	31	11
	14	55	19
	15	49	17
	16	67	24
	17	65	23
			
Patient sex	Female	192	68
	Male	92	32
			
Joint sessions (proportions)^a^	<1/3	43	15
	1/3–2/3	130	46
	>2/3	111	39
			
Self-rating^b^	No	163	57
	Yes	121	43
			
Healthcare profession^c^	Psychiatrist	169	60
	Psychologist	78	27
	Other profession	37	13
			
Time until diagnosis median	<1 week	179	63

^a^Proportion of sessions with patient and caregiver participating together (part sufficient); 1/3–2/3 = moderate.

^b^Montgomery Asberg Depression Rating Scale or Beck’s Depression Inventory, administered before session three.

^c^Psychiatrist ≥ 1 session (including non-specialist medical doctors); Psychologist ≥ 1 session (if no psychiatrist).

**Figure 5.**
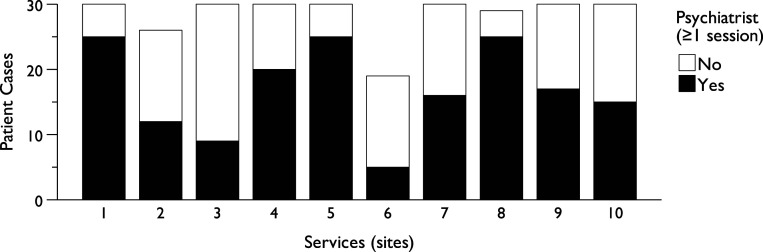
Distribution of service site characteristics (*n* = 284).

Patient age and sex were evenly distributed across sites, though variations were observed in age, joint sessions, self-ratings, psychiatrist sessions, and site distributions. The median number of cases per site was 27.5 (range: 19–30), as shown in Figure 5, which also presents the proportion of cases with at least one psychiatrist session. The median time to diagnosis was under one week (Quartile_3_ < 3, Percentile_90_ < 14), varying across sites and 0.5 weeks shorter with a psychiatrist involved. ICC values for interrater reliability of these characteristics ranged from .91 to 1.0 for average measures and .83 to 1.0 for single measures.

### Quality indicator components

Certain quality indicators tended to be documented together in medical records. Principal Component Analysis identified these co-occurrence patterns, grouping related indicators into components based on empirical associations, as shown by the standardised loadings in Table 2. Together, these six components explained 62% of the variance in indicator documentation. The first unrotated component accounted for 26%, but after rotation, each component explained 8%–15% of the variance. Proportions of explained variance are also detailed in Table 2. The mean indicator complexity was 1.9, supporting the adequacy of six components.

**Table 2.** Quality Indicator and Quality Component Correlations With Respective Explained Variances: Standardised Loadings From the Correlation Matrix of the Principal Component Analysis (*n* = 284).

Quality Components	RA	DD	DS	ID	ED	FL	Complexity
Indicator Correlations							
Risk Aspects (RA)							
Suicide risk assessment – structured	**.84**	.09	.19	−.06	−.06	−.21	1.3
Suicide risk assessment – graded	**.82**	−.24	.27	−.02	.04	.01	1.4
Suicide attempt	**.74**	.16	−.12	−.18	.10	.01	1.3
Non-suicidal self-injury	**.56**	−.04	**−.31**	.12	.05	.21	2.0
Recurrent thoughts of death/suicide	**.56**	.15	−.05	.27	−.17	**.30**	2.4
Insomnia/hypersomnia	**.49**	−.05	**.34**	**.30**	−.23	−.13	3.3
Substanceabuse	**.45**	.10	.09	.10	**.44**	−.15	2.5
Depression Distinct (DD)							
Episodicity – course of depression	.03	**.80**	−.10	.17	**−.33**	.03	1.5
Depressed/irritable mood	.10	**.71**	.01	−.13	.08	−.23	1.3
Significant weight/appetite change	−.03	**.61**	**.39**	.00	−.06	.03	1.7
Family psychiatric history	.02	**.57**	.08	−.13	.06	−.04	1.2
Depression Subtle (DS)							
Fatigue/loss of energy	.24	−.08	**.84**	−.22	−.09	.15	1.4
Diminished interest/pleasure	.00	**.32**	**.59**	.03	−.07	−.07	1.6
Diminished ability to think/concentrate	−.23	**.40**	**.47**	.07	.19	.16	3.2
Feelings of worthlessness/guilt	−.18	.22	**.34**	**.35**	.08	.15	3.8
Internalising Disorders (ID)							
Mania/ hypomania	.09	.08	−.19	**.90**	.09	−.13	1.2
Anxiety disorders/ OCD	−.11	−.10	−.07	**.81**	.02	.04	1.1
Externalising Disorders (ED)							
Disruptive behaviour disorders	−.06	.05	**−.45**	.17	**.86**	−.02	1.6
ADHD	.04	**−.30**	.23	−.04	**.85**	.09	1.4
Function & Life situation (FL)							
Peer relations	−.10	−.18	.05	.12	.02	**.78**	1.2
Schooling	−.02	−.04	**.36**	−.16	.07	**.72**	1.6
Family climate	.15	.21	−.12	**−.31**	−.02	**.57**	2.2
Diagnostic statement*	**.33**	.15	.13	.19	.16	.17	3.7

Component Correlations							
Risk Aspects							
Depression Distinct	**.39**						
Depression Subtle	.09	**.30**					
Internalising Disorders	.29	.29	**.40**				
Externalising Disorders	.16	.24	.16	.16			
Function & Life situation	.23	.27	.10	.20	.05		

Indicator variance explained	.15	.11	.11	.09	.08	.08	
Component variance explained	.24	.18	.17	.15	.13	.13	

*Note*. RA = Risk Aspects; DD = Depression Distinct; DS = Depression Subtle; ID = Internalising Disorders; ED = Externalising Disorders; FL = Function & Life situation; OCD = Obsessive Compulsive Disorder; ADHD = Attention Deficit Hyperactivity Disorder; Bold values = coefficients ≥ .3 or ≤ −.3; * Indicator without component affiliation; Component intercorrelation diagonal and upper triangle values are omitted.

The first component, named *Risk Aspects*, was associated with risk-related indicators, including two depressive symptoms, as shown in Table 2. The second component, named *Depression Distinct*, correlated with the core symptom depressed/irritable mood and depressive characteristics that may be more readily identified in clinical assessment, whereas the third, named *Depression Subtle*, was exclusively associated with more subtle, subjectively experienced symptoms, including the core symptom diminished interest/pleasure (anhedonia). The fourth and fifth components, named *Internalising Disorders* and *Externalising Disorders*, reflected differential or comorbid conditions, while the sixth, named *Function & Life situation*, pertained to functional status and living conditions. The diagnostic statement indicator showed weak correlations with all components, most notably with *Risk Aspects*, and was therefore not included in any component.

Some indicators were strongly associated with a single component, while others were linked to multiple components. For instance, weight/appetite and schooling indicators also tended to co-occur with indicators related to fatigue, anhedonia, and the ability to think/concentrate. Negative correlations, such as between disruptive behaviour disorders and *Depression Subtle*, indicate that these were less likely to be documented together. The complexity of indicator correlations across all components is presented in the last column of Table 2, where low complexity signifies independently documented indicators. Additionally, moderate correlations were observed among the first four components, which all reflect internalising characteristics.

### Quality indicator occurrences

**Figure 6.**
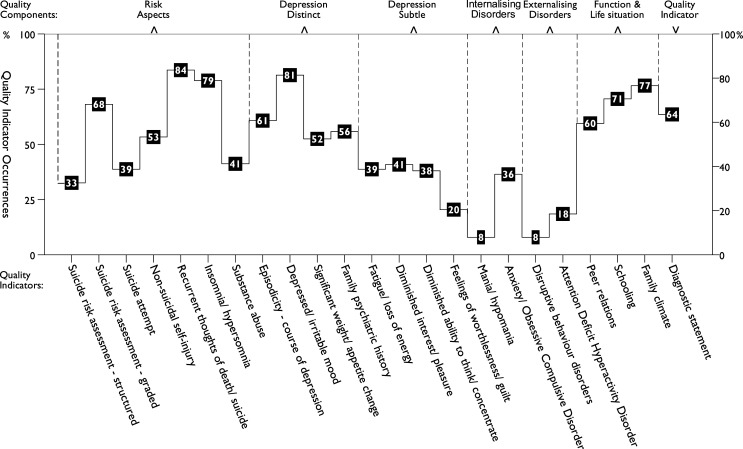
Occurrence of quality indicators in medical records, grouped by components from the principal component analysis (*n* = 284).

The documentation of quality indicators in medical records varied widely, ranging from 8% to 84%, with a mean of 49%. At least one affirmed core symptom of depression was fully documented in 220 records (77%; 95% *CI*: 72–82), though depressed/irritable mood was recorded twice as often as anhedonia. The most frequently documented indicators were recurrent thoughts of death/suicide (84%), depressed/irritable mood (81%), and insomnia/hypersomnia (79%), followed by family climate (77%) and schooling (71%). In contrast, structured suicide risk assessments (33%), feelings of worthlessness/guilt (20%), ADHD (18%), disruptive behaviour disorders (8%), and mania/hypomania (8%), were the least frequently documented indicators. Hence, both risk-related and depressive-symptom indicators were among the most but also the least frequently documented areas. An overview of each indicator’s occurrence, grouped by component, is shown in Figure 6, while summary measures and interrater reliability ICC values are detailed in Table 3.

**Table 3.** Occurrences of Quality Indicators and Components, Value Distributions, Correlations With Other Indicators, and Interrater Reliability (*n* = 284).

Quality Components Quality indicators	(Maximum per case)	Indicator occurrence summary					Value distributions			Value 2	Correlations with other	Interrater ICC
		%	95% *CI*	Mean	*SD*	Median	% 0	% 1	% 2	(Negated %)		Average (single)
Risk Aspects	(14)	57	[55–58]	7.93	3.60	8						
Suicide risk assessment – structured		33	[29–37]	0.65	0.84	0	**59**	17	24	N/A	.55	.83 (.70)
Suicide risk assessment – graded		68	[64–72]	1.36	0.86	2	25	14	**61**	N/A	.48	.89 (.80)
Suicide attempt		39	[35–43]	0.77	0.94	0	**58**	6	36	(57)	.47	.80 (.67)
Non-suicidal self-injury		53	[49–58]	1.07	0.92	1	39	16	**45**	(41)	.38	.74 (.59)
Recurrent thoughts of death/ suicide		84	[80–87]	1.67	0.67	2	12	9	**79**	(20)	.53	.81 (.68)
Insomnia/ hypersomnia		79	[75–82]	1.58	0.69	2	12	19	**69**	(16)	.43	.94 (.89)
Substance abuse		41	[37–45]	0.82	0.97	0	**57**	4	39	(80)	.51	.94 (.89)
Depression Distinct	(8)	63	[61–65]	5.01	2.26	5						
Episodicity – course of depression		61	[57–65]	1.21	0.91	2	32	14	**54**	N/A	.49	.80 (.67)
Depressed/ irritable mood		81	[78–84]	1.63	0.66	2	10	17	**73**	( 2)	.37	.95 (.91)
Significant weight/ appetite change		52	[48–57]	1.05	0.92	1	40	16	**44**	(26)	.56	.87 (.76)
Family psychiatric history		56	[52–60]	1.12	0.93	1	38	12	**50**	(10)	.39	.88 (.78)
Depression Subtle	(8)	35	[33–37]	2.76	2.24	2						
Fatigue/ loss of energy		39	[35–43]	0.77	0.84	1	**49**	24	27	( 3)	.44	.84 (.72)
Diminished interest/ pleasure		41	[37–45]	0.82	0.93	0	**54**	11	35	( 8)	.49	.87 (.77)
Diminished ability to think/ concentrate		38	[34–42]	0.76	0.83	1	**49**	26	25	(13)	.51	.84 (.73)
Feelings of worthlessness/ guilt		20	[17–24]	0.41	0.64	0	**67**	25	8	(17)	.44	.79 (.65)
Internalising Disorders	(4)	22	[20–25]	0.89	1.06	1						
Mania/ hypomania		8	[ 6–10]	0.16	0.53	0	**91**	2	7	(85)	.31	.94 (.89)
Anxiety disorders/ OCD		36	[33–42]	0.73	0.80	1	**49**	29	22	(13)	.31	.86 (.75)
Externalising Disorders	(4)	13	[11–15]	0.53	0.93	0						
Disruptive behaviour disorders		8	[ 6–10]	0.16	0.48	0	**88**	7	5	(38)	.14	.94 (.88)
ADHD		18	[16–22]	0.37	0.68	0	**75**	14	11	(18)	.24	.96 (.93)
Function & Life situation	(6)	69	[67–71]	4.13	1.43	4						
Peer relations		60	[55–64]	1.19	0.72	1	18	**45**	37	N/A	.27	.73 (.58)
Schooling		71	[67–74]	1.41	0.68	2	11	37	**52**	N/A	.38	.86 (.76)
Family climate		77	[73–80]	1.53	0.70	2	12	23	**65**	N/A	.25	.83 (.72)
Diagnostic statement*	(2)	64	[60–67]	1.27	0.69	1	14	**45**	41	N/A	.57	.74 (.59)
												
Indicator Index	(46)	49	[48–50]	22.52	7.56	23	**41**	19	40	Q_1–3_: 17–28	R: 1–41	α: .79 (.78)

*Note*. OCD = Obsessive Compulsive Disorder; ADHD = Attention Deficit Hyperactivity Disorder; * Indicator without component affiliation; Bold values = most frequent value; N/A = not applicable (negation impossible); Q_1–3_ = Quartile_1_ – Quartile_3_; R = Range; α = Cronbach’s alpha for internal consistency: Average measures (Standardised items).

Overall, subtle, subjectively experienced depressive symptoms were documented less often, as were evaluations of alternative diagnoses and comorbidities. Indicators were generally either missing or fully present, with partial documentation being rare. Among frequently documented indicators, like depressed/irritable mood, the absence of symptoms was rarely explicitly noted. In comparison, some rarely documented indicators were often negated, such as mania/hypomania, while others, like feelings of worthlessness/guilt, were rarely negated. Certain indicators were seldom documented alongside many others, as reflected in the weak correlation between disruptive behaviour disorders and other indicators in Table 3.

**Figure 7.**
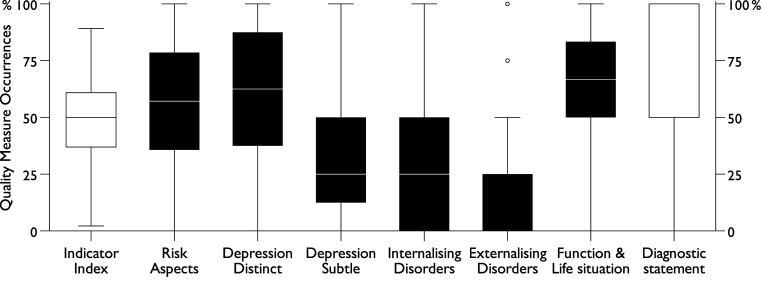
Standardised distributions of Indicator Index, components, and diagnostic statement (*n* = 284).

***Safe, Efficient, Effective, and** **Patient-** **centered** **care*****.** Indicators that tended to co-occur were grouped into empirically derived quality components, with documentation rates ranging from 13% to 69%, as presented in Table 3. The most frequently documented component was *Function & Life situation* (69%), followed by *Depression Distinct* (63%), and *Risk Aspects* (57%). In contrast, *Depression Subtle* (35%), *Internalising Disorders* (22%), and *Externalising Disorders* (13%) were less frequently documented. Among these components, *Risk Aspects* showed the widest variation in indicator documentation (33%–84%), followed by *Depression Distinct* (52%–81%) and *Internalising Disorders* (8%–36%). Conversely, *Function & Life situation* (60%–77%), *Depression Subtle* (20%–41%), and *Externalising Disorders* (8%–18%) exhibited more limited variation. These documentation patterns are detailed in Table 3 and Figure 6. To facilitate comparisons, Figure 7 illustrates standardised distributions of the Indicator Index, all quality components, and the diagnostic statement indicator.

### Quality indicator associations

***Equitable care*****.** Demographic and clinical predictors explained different proportions of documentation variance within the six quality indicator components, with associations detailed in Table 4. Model-I includes specified predictors – patient age, patient sex, joint sessions, self-rating, and psychiatrist sessions – as well as site predictors, whereas Model-II includes only the specified predictors. As indicated by the *R*^2^ values in Table 4, Model-I explained 12%–28% (adjusted 7%–24%) of the variance within the components, while Model-II explained 3%–17% (adjusted 1%–15%). *F*-values for each model per component are detailed in Table 4. Tolerance and variance inflation factor values ranged from .454 to .930 and 1.08 to 2.20 for Model-I, and from .935 to .997 and 1.00 to 1.08 for Model-II.

**Table 4.** Quality Component Associations With Demographic and Clinical Predictors (*n* = 284). Multiple Linear Regression: Model-I and Model-II.

Quality Components		Risk Aspects			Depression Distinct			Depression Subtle			Internalising Disorders			Externalising Disorders			Function & Life situation	
Component maximum		14.00			8.00			8.00			4.00			4.00			6.00		
Component mean		7.93			5.01			2.76			0.89			0.53			4.13		

Model-I:
Specified and Site^a^	*R* ^2^		.28	****		.20	****		.12	**		.16	****		.20	****		.16	****
Model-II:
Specified^a^	*R* ^2^		.17	****		.09	****		.05	**		.04	*		.08	***		.03	

Model-II: Parameter	Estimates	*b*	β		*b*	β		*b*	β		*b*	β		*b*	β		*b*	β	
Predictors:	(Intercept)	4.14	*		2.59	*		1.83			0.03			0.90			5.20	****	
Patient age	(≤12–17)	0.14	.06		0.10	.07		0.05	.03		0.05	.07		−0.04	−.07		−0.08	−.09	
Patient sex	(1 = male)	−0.95	−.12	*	−0.02	−.00		−0.22	−.05		−0.29	−.13	*	0.42	.21	***	−0.12	−.04	
Joint sessions^b^	(1 = >2/3)	0.12	.02		0.06	.01		−0.41	−.09		0.25	.11	†	−0.06	−.03		0.09	.03	
Self-rating^c^	(1 = yes)	1.18	.16	**	0.98	.22	***	0.87	.19	***	0.23	.11	†	0.39	.21	***	0.21	.07	
Psychiatrist^d^	(1 = yes)	2.52	.34	****	0.80	.17	**	0.07	.02		0.05	.02		−0.02	−.01		0.32	.11	†

Model-I:
Specified and Site^a^	*F*	7.32			4.68			2.49			3.76			4.65			4.65		
Model-II:
Specified^a^	*F*	11.22			5.18			2.95			2.29			5.07			1.49		

†*p* ≤ .07; *p ≤ .05; **p ≤ .01; ***p ≤ .001 = Bonferroni-corrected *p* = .05; ****p ≤ .0001 = Bonferroni-corrected *p* = .005; β = Part correlation; *F* = *F*-values from model ANOVA.

^a^Model-I = Specified predictors (age, sex, joint sessions, self-rating, and psychiatrist) and Site (service); Model-II = Specified predictors.

^b^Proportion of sessions with patient and caregiver participating simultaneously (part sufficient).

^c^Montgomery Asberg Depression Rating Scale (MADRS) or Beck’s Depression Inventory (BDI) administered before session three.

^d^Psychiatrist = At least one session with a psychiatrist or a non-specialist medical doctor during the assessment period.

The strengths of association between specified predictors in Model-II and each quality component is represented by the unstandardised (*b*) and standardised (β) regression coefficients in Table 4. Psychiatrist sessions, self-rating usage, and female patients were associated with better documentation for *Risk Aspects*. Self-ratings and psychiatrist sessions also predicted better documentation within *Depression Distinct*, as did self-ratings within *Depression Subtle*. Thus, self-rating was overall associated with better documentation of depressive characteristics. For *Internalising Disorders*, better documentation was predicted for female patients, whereas male patients and self-ratings were associated with better documentation for *Externalising Disorders*. Quality variance within *Function & Life situation* was partly explained by site predictors, representing unknown service-related factors, with no contribution from the specified predictors.

***Timely care*****.** The overall association between quality indicators and time until diagnosis was weakly negative, demonstrating that better documentation correlated with a shorter time to establish a depression diagnosis. Pearson correlation coefficients and corresponding *p*-values for the quality indicator measures are detailed in Table 5. More specifically, documentations of risk aspects, depressive symptoms and characteristics, and the diagnostic statement correlated with earlier diagnosis. In contrast, documentation of alternative diagnoses or comorbidities showed no correlation with diagnostic timeliness.

**Table 5.** Quality Measures Correlated to Time (Square Root Weeks) Until Diagnosis (*n* = 284).

Quality Measures	Pearson Correlation Coefficients (*r*)	*p*-values
Indicator Index	−.22	<.001
Components		
Risk Aspects	−.18	.002
Depression Distinct	−.19	.001
Depression Subtle	−.18	.003
Internalising Disorders	.07	.269
Externalising Disorders	.07	.217
Function & Life situation	−.14	.021
Indicator		
Diagnostic statement	−.17	.005

*Note*. Two-sided significance; *p* ≤ .006 = Bonferroni-corrected *p* = .05.

## Discussion

In this large national cross-sectional review of medical records from children and adolescents with depression, approximately half of the quality indicators were documented, with notable variation across assessment aspects, patient cases and service sites. Risk assessments varied significantly, with insufficient documentation of substance abuse, suicide attempts, and structured suicide risk evaluations, raising concerns about their validity and reliability. Depression core symptoms were unevenly documented, as depressed or irritable mood was recorded twice as often as anhedonia. Although mood and suicidal thoughts are subjectively experienced, their documentation did not correlate with that of more subtle internalising depressive symptoms, which were clearly less frequently recorded. Comorbidity assessments were particularly lacking, suggesting unstructured evaluations and limited use of structured diagnostic interviews. By contrast, function and life situation were generally well-documented, reflecting a focus on psychosocial aspects. Variance within these components was associated with patient sex (risk and comorbidity assessments), self-rating usage (risk and depressive-characteristics assessments), and psychiatrist sessions (risk assessments). Furthermore, unidentified service-related factors explained one-tenth of the variance, highlighting clear assessment inequities. Risk aspects showed the strongest associations with predictors, explaining over a quarter of the variance. Better overall documentation, particularly of depressive characteristics and the diagnostic statement, correlated with earlier diagnoses, whereas comorbidity documentation showed no association with diagnostic timeliness.

Few comparable studies exist, with only two using similar or larger samples (Hermens et al., 2015; Zima et al., 2005). Despite their heterogeneity, the approved proportions were broadly comparable to this study’s partially and fully documented indicators. The most frequently recorded indicators – recurrent thoughts of death, depressed mood, and sleep disturbances – aligned with similar studies, whereas structured suicide risk assessments, feelings of worthlessness or guilt, and comorbidities were less frequently documented. However, some aspects were documented as well as or better than in other studies, reflecting wide variability. Although no comparable study applied the IOM quality aims, this study’s indicator occurrences corresponded to Safe, Efficient, Effective, and Patient-centered care, while predictor associations and diagnostic timeliness addressed Equitable and Timely care. These IOM aims, detailed in Figure 1, are complementary and synergistic, though tensions and overlaps exist (IOM, 2001). Nonetheless, the results are further interpreted within this framework.

***Safe care*****.** Documentation of risk aspects was highly variable, potentially posing harm to patients. While three related indicators were frequently recorded, others were rarely noted. Given that suicide risk was graded moderate or high in nearly all cases, indicators such as structured suicide risk assessment, suicide attempt, and substance abuse should have been documented with similar frequency. This raises serious concerns about the validity and reliability of the suicide risk assessments, particularly in relation to more recent models of therapeutic risk assessment, formulation, and management (Hawton et al., 2022). Compared to previous studies, documentation of non-suicidal self-injury and graded suicide risk was similar (Ellis et al., 2019; Kramer et al., 2008), but recordings of substance abuse (Kramer et al., 2008; Zima et al., 2005), risk aspects in general (Pile et al., 2020), and structured suicide risk assessments (Zima et al., 2005) were notably more deficient. These discrepancies likely cannot be attributed to the more nuanced scale used in this study, indicating a need for quality improvement.

***Effective care*****.** Depressed or irritable mood was frequently documented, whereas anhedonia – the other core symptom of depression – was often missing, suggesting incomplete assessments and insufficient knowledge of diagnostic criteria and depressive symptoms. This indicates that many young patients with anhedonia alone may remain undiagnosed and, consequently, not receive appropriate treatment (Jensen-Doss & Weisz, 2008). Although at least one affirmed core symptom and episodicity were quite often recorded, a shortfall was evident given the diagnostic requirements for depressive episodes, aligning with findings of inadequate diagnostic criteria evaluations (Nakash et al., 2015). Family psychiatric history was documented less frequently than reported in other studies (Pile et al., 2020; Zima et al., 2005), despite its significance in diagnostic assessments and treatment planning (Thapar et al., 2022). Similarly, considerations of alternative explanations or comorbidities were less frequent (Ellis et al., 2019; Zima et al., 2005), with negative principal component analysis associations for externalising disorders suggesting a tendency to neglect these while focusing on certain depressive characteristics. Whether documentation likelihood was influenced by the order of clinical interview topics is unclear, though this would align with “search satisficing” diagnosticians missing diagnoses (Jensen-Doss et al., 2014). Infrequent comorbidity considerations may reflect a lack of structured diagnostic interviews, contributing to suboptimal diagnostic procedures. Other studies did not include indicators for specific symptoms or comorbidities. Although not directly comparable, this study’s mean indicator occurrence exceeded the reported use of semi-structured interviews in another study (Hermens et al., 2015), while diagnostic statement occurrences aligned with findings on severity and functioning in similar studies (Ellis et al., 2019; Hermens et al., 2015).

***Efficient care*****.** Indicators were mostly either missing or fully documented (rarely partly present), indicating an “all-or-nothing” documentation tendency. The least frequently documented indicators – suicide attempt, substance abuse, and mania/hypomania – were generally negated when recorded. However, low occurrences of comorbidity indicators cannot be interpreted as undocumented negations, given depression’s strong association with other disorders (Avenevoli et al., 2015). Whether negations were less frequently recorded remains unclear but is likely. In services with low continuity or involving many professionals in LEAD-like procedures, this could lead to uncertainty about what has been assessed, resulting in repeated questioning, wasted resources, and exhausting patients and caregivers. Thus, documenting both presence and absence of symptoms is crucial. In contrast to the observed variability, contributing to independently occurring components, properly documented structured diagnostic interviews would yield no distinguishable components, which ought to be the ultimate goal. However, the addition of fully structured diagnostic interviews may be neither necessary nor justified (Sayal et al., 2025).

***Patient-** **centered** **care*****.** The limited documentation of more subtle, subjectively experienced symptoms underscores the need to prioritise adolescents’ self-reports, which are crucial for assessing depression and suicidal behaviour (De Los Reyes et al., 2015; Lewis et al., 2014), as well as for developing personalised clinical formulations and treatment plans (Herrman et al., 2022; Krause et al., 2023). This aligns with research emphasising the value of integrating adolescents’ perspectives in psychotherapy (Housby et al., 2021). As no similar study used detailed indicators for depressive characteristics, direct comparisons are not possible. However, other aspects of patient-centeredness were adequately addressed in this study, with function and life situation generally well-documented. This study reported better documentation for schooling and similar levels for peer relations and family climate compared to previous research (Ellis et al., 2019; Zima et al., 2005), all related to treatment outcomes most important to young people and thus highly relevant in patient-centered care (Krause et al., 2023).

***Equitable care*****.** Quality component associations with predictors revealed significant inequities, though some were modest. Unknown service-related factors accounted for one-tenth of the variance, alongside self-rating and psychiatrist sessions, confirming regional disparities noted in a national inventory using broader indicators (Socialstyrelsen, 2019). This highlights the need for guideline implementation and educational efforts, while also emphasising local responsibility for healthcare quality. The strongest association was between psychiatrist sessions and better documentation of risk aspects, possibly because psychiatrists handle more severe cases. However, risk assessments should be comprehensive for all depression patients. Notably, psychiatrist sessions did not predict better documentation of more subtle depressive symptoms or comorbidities.

Self-rating was clearly associated with better documentation of depressive symptoms, suggesting it may enhance diagnostic effectiveness through patient-centeredness, emphasising the importance of self-reports (Lewis et al., 2014). Unexpectedly, self-rating was also associated with externalising disorders, indicating that its structure might improve documentation beyond depression. However, associations with self-rating and psychiatrist sessions might partly reflect other clinical factors. Documentation estimates for risk aspects and internalising disorders were better for female patients, while estimates for externalising disorders were better for males. These differences raise concerns about gender bias, given the higher ADHD risk in females with depression (Avenevoli et al., 2015). No clear associations emerged for joint sessions, likely due to caregiver participation in all cases.

***Timely care*****.** Reducing diagnostic delays is vital for effective treatment and recovery (Jensen-Doss & Weisz, 2008), as is identifying comorbidities associated with worse outcomes and requiring treatment adjustments (Thapar et al., 2022). Comprehensive documentation, particularly of depressive features and diagnostic statements, was associated with timelier diagnoses, whereas documenting comorbidities showed no correlation. Their infrequent recordings, may thus have delayed detection of other psychiatric disorders, underscoring the need of thorough evaluations. Assuming that earlier diagnoses lead to faster treatment and recovery, these findings support associations between better documentation and improved patient outcomes, thereby strengthening the construct validity of the quality measures. It also aligns with specific depression assessment standards (NICE, 2013). Nonetheless, it should be noted that most patients were diagnosed within a week, and the timeliness of treatment initiation was not examined.

### Clinical implications

The substantial variability in documentation highlights the need for more structured approaches to improve clinical practice. Greater attention is required for psychiatric aspects of depressive disorders, comorbidities, and patient safety, particularly in suicide assessments, which must account for key factors such as suicide attempts, substance abuse, and self-harming behaviour (Hawton et al., 2022). Implementing systematic diagnostic processes grounded in established criteria for depression and other psychiatric disorders could help address these concerns while also improving the evaluation of subtle internalising symptoms, alternative diagnoses, and comorbidities (Jensen-Doss et al., 2014; Nakash et al., 2015). Clinicians should also prioritise adolescents’ self-reports, which are crucial for assessing and treating internalising disorders and suicide risk (De Los Reyes et al., 2015; Hawton et al., 2022; Housby et al., 2021; Krause et al., 2023; Lewis et al., 2014). Use of self-rating scales may further support this process.

Additionally, raising awareness of gender bias among diagnosticians could help reduce assessment inequities. Underreporting of negations warrants attention, particularly in low-continuity services and those involving multiple professionals. More structured diagnostic procedures may address these issues, enhancing diagnostic validity and reliability. Systematic assessments do not necessarily require fully structured diagnostic interviews, as flexibility may be preferable. However, incorporating a supportive structure, along with sufficient training, is recommended to improve assessment quality.

### Strengths and limitations

This study used a large, nationally representative sample from diverse CAMHS, with patient age and sex distributions aligning with known prevalence (Shorey et al., 2021). Although patients could opt out, few chose to do so. Key strengths included the use of a three-step quality scale, empirically based components, and an established overarching framework (IOM, 2001). The guideline (Jarbin et al., 2014), along with the DSM-5 and K-SADS (APA, 2013; Jarbin et al., 2017), supported the content validity of the indicators. Construct validity was confirmed through the internal consistency of all quality indicators, as well as the principal component analysis, regressions, and correlation analyses linking quality measures to potential patient outcomes (Wensing et al., 2020). Validity and reliability were further demonstrated by respectable to very good MRR interrater reliability (DeVellis, 2017), including investigator re-reviews. These elements provided nuanced insights into assessment practices that might otherwise remain undetected.

***Limitations.*** MRR proxy measurements have known limitations (Gearing et al., 2006; Vassar & Holzmann, 2013; Worster & Haines, 2004). The absence of indicators in medical records does not necessarily imply omitted processes, potentially affecting measurement reliability. Actual assessments were likely more comprehensive than reflected by indicator occurrences, as few negations were documented. However, diagnostic processes and suicide risk assessments should not be among the less frequently documented phenomena. Participation was voluntary, introducing potential selection bias, as participating services may have had greater quality improvement needs or ambitions. Case selection relied on diagnostic codes from administrative systems, which may not represent all true cases of depression, possibly explaining the high psychiatrist involvement. Negated issues were coded separately only when fully present, although partly present negations were likely rare. One depressive symptom – psychomotor agitation or retardation – was not included. As the list of comorbidities was not exhaustive, these recordings might be underestimated.

Regression and correlation analyses involved multiple comparisons but were primarily exploratory. Therefore, multilevel analyses and extensive validity or reliability assessments were not conducted. Due to slight positive skew in the *Externalising Disorders* component, preliminary analyses using its natural logarithm were performed, revealing non-significant *F*-values but a positive association for male patients. Predictors did not include socioeconomic or family-related factors, which might have provided further insights. Structured diagnostic interviews, clinician experience or training level, and other service factors beyond self-rating and profession were also not examined. Their impact may be encompassed within the unknown service-related factors.

***Generalisability*****.** Findings from this study are likely applicable to other outpatient CAMHS settings. The combination of evidence-based indicators, the IOM framework, and an empirical methodology strengthens external validity and enhances comparability. However, the voluntary participation of services and specific regional characteristics in Sweden may limit full generalisability. Nonetheless, the study’s methodology provides a robust approach to conceptualising, measuring, and analysing healthcare quality with reasonable validity and reliability. It not only addresses existing knowledge gaps but may also contribute to evolving methodologies. Indicators developed in this study should be transferable to medical records in other healthcare systems. They also align with calls for better data sources to monitor this area and may support more proactive suicide prevention. The broad relevance of the predictors could help formulate future hypotheses and guide research directions.

### Conclusions

This study revealed considerable variability in how depression assessments were documented across assessment aspects, patients, and services. On average, only half of the recommended areas were properly recorded. The use of a continuous quality scale may have provided more nuanced insights, highlighting substantial differences in documentation. Depressed mood, suicidal thoughts, and sleep disturbances were among the most frequently documented aspects, along with patient functioning and living conditions. In contrast, more subtle depressive symptoms and evaluations of other mental health conditions were often overlooked, as were some of the most crucial suicide risk factors. These gaps underscore the key role of systematic assessment processes and additional clinician training.

Identifying documentation patterns before implementation interventions may help develop tailored strategies for enhancing guideline adherence, aligning with calls for new implementation approaches. The association between better documentation and earlier diagnoses indicates the importance of documentation quality for patient outcomes. Service-related factors contributed significantly to variance and warrant further investigation. Nevertheless, these findings suggest that improving depression assessment quality requires a tailored approach rather than a “one-size-fits-all” strategy.

## Supplemental Material

Supplemental Material - Quality of Depression Assessments in child and adolescent psychiatry: Findings from a nationwide Swedish outpatient medical record review Supplemental Material for Quality of Depression Assessments in child and adolescent psychiatry: Findings from a nationwide Swedish outpatient medical record review by Susanne Remvall, Anna Helena Elisabeth Santesson, Martin Bäckström, Björn Hofvander and Håkan Jarbin in Clinical Child Psychology and Psychiatry.

## Data Availability

The dataset generated and analysed during this study is available from the corresponding author on reasonable request.*
