# Supplementary material for: Quality of depression assessments in child and adolescent psychiatry: Findings from a nationwide Swedish outpatient medical record review
Source: Clin Child Psychol Psychiatry. 2025 Jun 24;30(4):904–30. doi: 10.1177/13591045251341919 (PMC13066466; doi:10.1177/13591045251341919)
Supplement: Supplemental Material - Quality of Depression Assessments in child and adolescent psychiatry: Findings from a nationwide Swedish outpatient medical record review [file sj-pdf-1-ccp-10.1177_13591045251341919.pdf]

## Supplemental material

**Table S1.** Medical Record Review Protocol: Variables, Abstraction Codes, Requirements, and Values for Analysis.

| Quality indicators | Abstraction codes | Coding requirements | Values |
|---|---|---|---|
| Depressed or irritable mood<br>(core symptom) | 1=present<br>2=partly present<br>3=negated<br>9=missing | 1=referring to mood<br>2=reactive sadness mentioned | 2<br>1<br>2<br>0 |
| Diminished interest or pleasure/ anhedonia<br>(core symptom) | 1=present<br>2=partly present<br>3=negated<br>9=missing | 1=reduced activities due to anhedonia described<br>2=reduced activities mentioned, anhedonia not clearly described | 2<br>1<br>2<br>0 |
| Significant weight or appetite change<br>(additional symptom) | 1=present<br>2=partly present<br>3=negated<br>9=missing | 1=changed appetite or weight<br>2='bad food intake' mentioned but not specified | 2<br>1<br>2<br>0 |
| Insomnia or hypersomnia<br>(additional symptom) | 1=present<br>2=partly present<br>3=negated<br>9=missing | 1=clearly described sleep issues: insomnia, wake-ups, too little or much<br>2='sleeping problems' or 'bad sleep' mentioned but not specified<br>3='good sleep' not specified | 2<br>1<br>2<br>0 |
| Fatigue or loss of energy<br>(additional symptom) | 1=present<br>2=partly present<br>3=negated<br>9=missing | 1=new kind of fatigue or loss of energy nearly every day described<br>2='tiredness' (not loss of energy) mentioned but not specified | 2<br>1<br>2<br>0 |
| Feelings of worthlessness or excessive guilt<br>(additional symptom) | 1=present<br>2=partly present<br>3=negated<br>9=missing | 1=accentuated feelings of worthlessness or inappropriate guilt<br>2=self-criticism mentioned but not specified | 2<br>1<br>2<br>0 |
| Diminished ability to think or concentrate, or indecisiveness<br>(additional symptom) | 1=present<br>2=partly present<br>3=negated<br>9=missing | 1=new kind of reduced ability to think or concentrate clearly described<br>2=concentration problems mentioned but not specified | 2<br>1<br>2<br>0 |
| Recurrent thoughts of death or suicidal ideation<br>(additional symptom) | 1=present<br>2=partly present<br>3=negated<br>9=missing | 1=suicide intention, intensity, and frequency, clearly described<br>2='thoughts of death' or similar mentioned (no specified level) | 2<br>1<br>2<br>0 |
| Episodicity – course of depression | 1=present<br>2=partly present<br>9=missing | 1=depressive symptoms onset and changed function clearly described<br>2= mood and function appears worsened, although vaguely described | 2<br>1<br>0 |
| Family psychiatric history | 1=present<br>2=partly present<br>3=negated<br>9=missing | 1=information noted under specific medical record heading, 'Heredity'<br>2=vaguely mentioned ('parent not feeling well') under another heading | 2<br>1<br>2<br>0 |
| Mania or hypomania – bipolar disorder<br>(differential diagnosis) | 1=present<br>2=partly present<br>3=negated<br>9=missing | 1=clear comments on mania, hypomania, or episodic 'increased energy'<br>2=comments on reactively 'elevated mood', not clearly episodic | 2<br>1<br>2<br>0 |
| Anxiety disorders or OCD<br>(co-existing or differential diagnosis) | 1=present<br>2=partly present<br>3=negated<br>9=missing | 1=comments clearly addressing at least one anxiety syndrome or OCD<br>2=anxiety in general addressed (anxiety as a symptom, not syndrome) | 2<br>1<br>2<br>0 |
| ADHD<br>(co-existing or differential diagnosis) | 1=present<br>2=partly present<br>3=negated<br>9=missing | 1=clear consideration of ADHD symptoms prior to depressive episode<br>2=school/concentration problems with no distinction from depression | 2<br>1<br>2<br>0 |
| Disruptive behaviour disorders<br>(co-existing or differential diagnosis) | 1=present<br>2=partly present<br>3=negated<br>9=missing | 1=behaviour disorder (ODD or CD), clearly addressed or considered<br>2=behaviour problems/messiness or similar mentioned, not specified | 2<br>1<br>2<br>0 |

*(continued)*

**Table S1.** (continued)

| Quality indicators | Abstraction codes | Coding requirements | Values |
|---|---|---|---|
| Substance abuse<br>(patients >12 years) | 1=present<br>2=partly present<br>3=negated<br>8=not applicable<br>9=missing | 1=use of tobacco, alcohol, or narcotic substances clearly described<br>2=mentioned without any specification or 'passing by'<br>8=screening not mandatory for children ≤12 years | 2<br>1<br>2<br>2<br>0 |
| Family climate<br>(function and life situation<br>– maintaining/protective) | 1=present<br>2=partly present<br>3=negated<br>9=missing | 1=family climate investigated before treatment (depression-related)<br>2=briefly mentioned, without negations, missing in final assessment | 2<br>1<br>2<br>0 |
| Peer relations<br>(function and life situation<br>– maintaining/protective) | 1=present<br>2=partly present<br>3=negated<br>9=missing | 1=current peer relations compared to before depressive episode<br>2=briefly mentioned, without description of change due to depression | 2<br>1<br>2<br>0 |
| Schooling<br>(function and life situation<br>– maintaining/protective) | 1=present<br>2=partly present<br>3=negated<br>9=missing | 1=school functioning compared to before depressive episode<br>2=briefly mentioned, without description of change due to depression | 2<br>1<br>2<br>0 |
| Suicide attempt<br>(ever) | 1=present<br>2=partly present<br>3=negated<br>9=missing | 1=suicide attempt mandatory to investigate at first session<br>2=suicide attempt described but not when, method, or seriousness<br>3=clearly asked and negated | 2<br>1<br>2<br>0 |
| Non-suicidal self-injury<br>(ever) | 1=present<br>2=partly present<br>3=negated<br>9=missing | 1=non-suicidal self-injury clearly described: period, intensity and frequency<br>2=non-suicidal self-injury mentioned but not when, the extent, or current situation | 2<br>1<br>2<br>0 |
| Suicide risk assessment<br>– graded<br>(low–moderate–high) | 1=present<br>2=partly present<br>9=missing | 1=graded as low, moderate or high, including motivation<br>2=commented without grading or graded without motivation<br>9=missing | 2<br>1<br>0 |
| Suicide risk assessment<br>– structured: including<br>important risk factors<br>(if risk graded >low) | 1=present<br>2=partly present<br>8=not applicable<br>9=missing | 1=structured suicide risk assessment, including risk factors<br>2=assessed without structured consideration of risk factors<br>8=not applicable when suicide risk had been graded as low<br>9=missing | 2<br>1<br>2<br>0 |
| Diagnostic statement<br>– summative assessment | 1=present<br>2=partly present<br>9=missing | 1=diagnosis with motivation, severity, and other diagnoses considered<br>2=diagnosis stated without severity, with other disorders considered<br>9=position on diagnosis is missing (repeated anamnesis) | 2<br>1<br>0 |

| Descriptive factors | Abstraction values | Value requirements |
|---|---|---|
| Patient age | ≤ 17 years | Number of full years at first session |
| Patient sex | 0=Female<br>1=Male | Sex (as per social security number) |
| Joint sessions | 1=Low <1/3<br>2=Moderate 1/3–2/3<br>3=High >2/3 | Proportion of sessions with patient and caregiver participating simultaneously – part of session sufficient |
| Self-rating scale<br>for depression | 0=Not used<br>1=Used | 0=Not used as described below or other scale used<br>1=MADRS or BDI administered no later than before the third session |
| Profession of<br>diagnostician | 1=Psychiatrist<br>2=Psychologist<br>3=Other profession | 1=Psychiatrist or non-specialist doctor, at least one session<br>2=Psychologist, unspecified, at least one session (if no psychiatrist)<br>3=Other profession (if no psychiatrist or psychologist participation) |
| Site | 1–10 | Child and Adolescent Mental Health Service site number |
| Time until diagnosis | ≥ 0 | Number of full weeks from first session until depression diagnosis |

*Note.* OCD = Obsessive Compulsive Disorder; ADHD = Attention Deficit Hyperactivity Disorder; ODD = Oppositional Defiant Disorder; CD = Conduct Disorder; MADRS = Montgomery Asberg Depression Rating Scale; BDI = Beck's Depression Inventory.
